# Hope and challenges working at the frontiers of medical knowledge

**DOI:** 10.4102/sajid.v37i2.405

**Published:** 2022-02-14

**Authors:** Graeme Meintjes

**Affiliations:** 1 Department of Medicine, Faculty of Health Sciences, University of Cape Town, Cape Town, South Africa; 2 SARChI Chair of Poverty-related Infections, Faculty of Health Sciences, University of Cape Town, Cape Town, South Africa; 3 Wellcome Centre for Infectious Diseases Research in Africa (CIDRI-Africa), Institute of Infectious Disease and Molecular Medicine, University of Cape Town, Cape Town, South Africa

I am currently the Second Chair and Deputy Head of the Department of Medicine at the University of Cape Town (UCT) and Groote Schuur Hospital. I also hold the SARChI Chair of Poverty-related Infections and lead a research group in the Wellcome Centre for Infectious Diseases Research in Africa (CIDRI-Africa) located within UCT’s Institute of Infectious Disease and Molecular Medicine.

*Why did I choose ID?* My Infectious Disease (ID) specialisation journey began as a medical registrar in 2001 and 2002. I spent several rotations in those years working at GF Jooste Hospital, which was the only public sector hospital on the Cape Flats at that stage serving over 1 million people. It was the height of the AIDS epidemic; however, antiretroviral therapy (ART) was not yet available in the public sector. Every day in the wards, we encountered tragedy; young people in distress, worsened by the stigma and hopelessness surrounding the disease, dying of HIV complications despite our best efforts to treat their opportunistic infections. Even for those patients who did respond to treatment, we knew that it was temporary, and they would return with another opportunistic infection. At the same time, the promise of ART was in the air. Some patients were accessing ART through trials or finding money to purchase privately; however, it was still too expensive for most. Then came the first pilot ART projects in our communities – established in Khayelitsha by Médecins Sans Frontières and at Gugulethu clinic by the Desmond Tutu HIV Centre. We witnessed the remarkable effects of ART in restoring health; however, in our hospital, we also encountered novel clinical problems: immune reconstitution inflammatory syndrome (IRIS), stauvudine side effects like lactic acidosis and many others. Some of the clinical presentations had not previously been described. The significant interaction of tuberculosis (TB) and HIV in the context of ART scale-up resulted in unique challenges. The tragedy became mixed with a sense of hope, but also the intellectual challenge of working at the frontiers of medical knowledge.

These realities and challenges drew me to specialise in ID and provided the context of my training and launch of my research career. I carried out ID clinical training under Gary Maartens’ mentorship, with Kevin Rebe as a co-trainee in 2003–2004. This was before ID’s recognition as a sub-specialty, and we later received ‘grandfather’ accreditation in 2007. Our training was split between Groote Schuur and GF Jooste. Given the contemporary realities, there was a very strong HIV and TB focus in our training.

**FIGURE 1:**
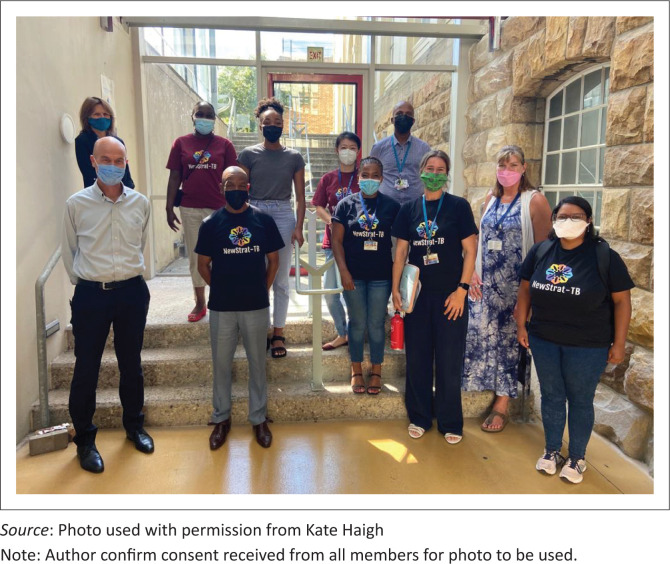
Graeme Meintjes (front row, far left) with members of the clinical research team currently conducting the NEW STRAT-TB trial at Mitchells Plain and Khayelitsha Hospitals, which is evaluating novel strategies for treating patients admitted with disseminated HIV-associated tuberculosis.

I then undertook a PhD, co-supervised by Robert Wilkinson and Gary Maartens, which involved treatment and pathogenesis studies of TB–IRIS using the clinical problems we were facing in our wards as the starting point. The first trial I led evaluated prednisone for the treatment of TB–IRIS. Since then, I have developed and led a research group that has conducted clinical trials (Figure 1), cohort and pathogenesis studies. The main focus of our research is conditions affecting patients with advanced HIV, including TB, cryptococcal meningitis and IRIS, often as part of multicentre collaborations. Some of our findings have impacted national and international guidelines. I have derived great fulfilment combining clinical practice and research as an ID specialist.

*What advice would I offer junior colleagues wanting to pursue ID research?* Firstly, try to secure a period of time to be able to concentrate predominantly on research, allowing one to develop analytical and writing skills. This may involve undertaking a PhD or a dedicated research period as part of a larger research group. Secondly, define a niche area to focus your research on, ensuring that you are carrying out unique research that can best be performed in your setting. This hopefully means your research will be of international interest, not simply reproducing findings from other settings. Thirdly, stay clinically active as that is where clinical research questions are best conceived and crafted into studies. Fourthly, find mentors who can guide you in where to focus your energies and time and define relevant, impactful, and feasible research questions. Fifthly, pay attention to establishing strong relationships with peers within the ID specialty and beyond. Networks you build in this way can provide important support and provide opportunities for collaborative research, sounding out new ideas and stimulating discussions. We all have different areas of strength.

Another fulfilling aspect of ID has been the opportunity to contribute to clinical guidelines at multiple levels. I encourage junior colleagues to take opportunities to sit on guideline committees and prepare by reading extensively around the topic so as to maximise their contributions. Decision making in guideline development is different from decision-making for individual patient care and an important aspect of an ID specialist’s role. Guideline panels also provide a unique opportunity to learn from colleagues.

